# Multi-Effects of Acute Salinity Stress on Osmoregulation, Physiological Metabolism, Antioxidant Capacity, Immunity, and Apoptosis in *Macrobrachium rosenbergii*

**DOI:** 10.3390/antiox12101836

**Published:** 2023-10-07

**Authors:** Yakun Wang, Huarong Li, Jie Wei, Kunhao Hong, Qiaoyan Zhou, Xiaoli Liu, Xiaoyou Hong, Wei Li, Chao Liu, Xinping Zhu, Lingyun Yu

**Affiliations:** 1 Key Laboratory of Tropical & Subtropical Fishery Resource Application & Cultivation of Ministry of Agriculture and Rural Affairs, Pearl River Fisheries Research Institute, Chinese Academy of Fishery Sciences, Guangzhou 510380, China; wangyk@prfri.ac.cn (Y.W.); lihuarong@prfri.ac.cn (H.L.); zjweijie@prfri.ac.cn (J.W.); hongkunhao@prfri.ac.cn (K.H.); zhouqiaoyan@prfri.ac.cn (Q.Z.); liuxl@prfri.ac.cn (X.L.); hxy@prfri.ac.cn (X.H.); liwei@prfri.ac.cn (W.L.); liuchao@prfri.ac.cn (C.L.); 2 College of Fisheries and Life Science, Shanghai Ocean University, Shanghai 201306, China

**Keywords:** giant freshwater prawn, euryhalinity, reactive oxygen species, qPCR, TUNEL assay

## Abstract

Salinity stress can trigger a series of physiological changes. However, the mechanism underlying the response to acute salinity stress in *Macrobrachium rosenbergii* remains poorly understood. In this study, osmoregulation, physiological metabolism, antioxidant capacity, and apoptosis were examined over 96 h of acute salinity stress. Hemolymph osmolality increased with increasing salinity. After 48 h of salinity exposure, the glucose, triglycerides, total protein, and total cholesterol contents in two salinity stress groups (13 and 26‰ salinity) were significantly lower than those in the 0‰ salinity group. The highest levels of these parameters were detected at 6 h; however, superoxide dismutase (SOD), total antioxidant capacity (T-AOC), and malondialdehyde (MDA) were the lowest at 96 h in the 13‰ salinity group. The activity of immunity-related enzyme alkaline phosphatase (AKP) showed a decreasing trend with increasing salinity and remained at a low level in the 26‰ salinity group throughout the experiment. No significant differences were observed in aspartate aminotransferase (AST), alanine aminotransferase (ALT), or lysozyme (LZM) among the three treatments at 96 h. After 96 h of salinity treatments, the gill filament diameter significantly decreased, and a more pronounced terminal deoxynucleotidyl transferase dUTP nick end labeling (TUNEL)-positive signal was detected in the 13‰ and 26‰ groups compared to that in the 0‰ group. Expression levels of apoptosis-related genes, including Cysteine-aspartic acid protease 3 (*Caspase 3*), Cysteine-aspartic acid protease 8 (*Caspase 8*), Cytochrome c (*Cyt-c*), tumor suppressor gene (*P53*), Nuclear factor kappa-B (*NF-κB*), and B cell lymphoma 2 ovarian killer (*Bok*) were significantly higher in the 26‰ salinity group than in the other groups at 24 h, but lower than those in the 0‰ salinity group at 96 h. *Cyt-c* and *P53* levels exhibited a significantly positive relationship with MDA, AST, and LZM activity during salinity stress. In the 13‰ salinity group, *Bok* expression was significantly correlated with SOD, T-AOC, AKP, acid phosphatase, and LZM activity, whereas in the 26‰ group, the AST content was positively correlated with *Caspase 8*, *Cyt-c*, and *P53* expression. A significant negative relationship was observed between *Caspase 3* expression and catalase (CAT) activity. These findings provide insight into the mechanisms underlying the response to acute salinity stress and will contribute to improving *M. rosenbergii* aquaculture and management practices.

## 1. Introduction

Salinity is regarded as a crucial environmental factor that influences various aspects of aquatic animal life, including reproduction, growth, development, and survival [1,2,3]. Some crustacean species, such as decapods, migrate from freshwater to brackish estuaries before attaining sexual maturity for mating, spawning, and hatching [2,4,5,6]. Crustaceans frequently encounter considerable salinity variations throughout their migration and adjust the hemolymph ion concentration, osmotic pressure, and enzyme activity associated with metabolism to maintain ion balance within their cells and adapt to the changing salinity of their environment, ensuring the maintenance of a stable internal environment and normal physiological metabolism [3]. Therefore, the mechanism of hemolymph osmoregulation has become a hotspot for studying the response of crustaceans to salinity stress [7,8,9].

Hemolymph and blood osmolality are commonly measured to assess the osmoregulatory capacity of aquatic animals. At salinities below and above the isosmotic concentration, the hemolymph becomes hyperosmotic and hypo-osmotic, respectively, in the surrounding medium [10]. Na^+^-K^+^-ATPase (NKA) is typically an essential enzyme that plays a crucial role in regulating ions and maintaining osmotic pressure balance within intra- and extracellular fluids [11]. Numerous studies have indicated that NKA activity and mRNA expression levels are significantly upregulated in salt-transporting tissues, such as hemolymph and gills, when exposed to salinity challenges [12,13].

The hepatopancreas is an important digestive gland in crustaceans that plays a critical role in physiological metabolism and antioxidant defense in response to environmental stress [14]. Under stressful conditions, such as salinity challenges, the metabolic rate increases to compensate for physiological changes. Therefore, acclimatization to salinity stress requires substantial energy consumption through catabolism of sugars and fats [15]. Blood glucose (GLU) and energy reactions modulate osmotic pressure balance, and lipid metabolism differs under varying salinity conditions [16]. Additionally, free amino acids in the hemolymph increase during salinity stress [17,18]. Thus, the metabolism of GLU, lipids, and proteins thus plays vital roles in responding to salinity challenges.

Furthermore, these stress-induced challenges often lead to an increase in reactive oxygen species (ROS), ultimately inducing oxidative stress, which adversely affects crustaceans [19,20]. Many crustacean species typically develop an immune response, such as salinity fluctuations, to adapt to changing environments. However, if environmental stress exceeds an organism’s tolerance, free radical production and removal in the body become imbalanced, triggering tissue cell apoptosis, and this can be detected through histological and molecular biological methods. Previous studies have found that low or high salinity typically results in varying degrees of apoptosis [21,22].

The giant freshwater prawn (*Macrobrachium rosenbergii*) is an economically important cultured species worldwide. It grows and develops in freshwater and migrates to estuaries for reproduction, where water of a specific salinity level is necessary for larval metamorphosis and hatching. During migration, this species develops high tolerance to a wide salinity range [23]. The ability to adapt to metabolic and physiological conditions and cope with environmental salinity changes is crucial for growth and survival. Multiple studies have reported that salinity changes can lead to delayed metamorphosis, decreased activity, and disease occurrence, potentially affect feeding, survival, and growth during the breeding period or larval stage [24,25,26,27,28,29,30]. However, there is limited knowledge regarding the physiological, histological, and molecular changes that occur in subadult *M. rosenbergii* as it transitions from freshwater to brackish water. Therefore, further investigations are needed to explore the mechanisms underlying the response to acute salinity stress.

Consequently, this study aimed to explore the response of individual *M. rosenbergii* to acute salinity stress by examining their histological features, physiological metabolism, antioxidant defense, immunity, and apoptosis. The results will contribute to advancing the current understanding of the salt tolerance mechanisms in this species, potentially leading to improved survival rates when cultivated in high-salinity water. Moreover, these findings will provide valuable guidance for the production of *M. rosenbergii*.

## 2. Materials and Methods

### 2.1. Experimental Materials and Design

The experiment was conducted in glass aquaria (60 × 50 × 50 cm) at the Pearl River Fisheries Research Institute, CAFS. A total of 360 healthy cultured prawns with an average body weight of 12.49 ± 3.08 g were obtained from Liantang Town, Zhaoqing City, Guangdong Province. Prior to the experiment, the prawns were acclimatized in an indoor aquarium for 5 days during which the water temperature was maintained at 30 ± 0.5 °C, dissolved oxygen levels were maintained at 6 mg/L, and the pH ranged from 7.0 to 8.0. The prawns were fed a commercial artificial compound diet twice daily at 8:00 a.m. and 5:00 p.m., and residues and excreta were removed through regular water changes. Before the start of the experiment, the prawns were fasted for 24 h.

Following acclimation, the prawns were randomly assigned to three treatment groups: 0, 13, or 26‰ salinity. The salinity was measured by using a salinometer (Thermo Eutech, Carlsbad, CA, USA). There were 3 treatments and a total of 9 tanks, with 40 individuals per tank. The control group was maintained in freshwater (0‰ salinity), and the higher-salinity groups (13‰ and 26‰) were achieved by diluting the seawater stock solution with fully aerated dechlorinated tap water.

### 2.2. Sample Collection

When the experiment commenced, six prawns (two per tank; sample sizes also apply to the sections that follow) from each treatment group were randomly selected and anesthetized within an ice bath for tissue collection at five time-points (6, 12, 24, 48, and 96 h after the treatment started).

The hemolymph samples were taken from the base of the prawn’s fifth walking limb using a 1 mL syringe washed with heparin sodium salt, placed on ice, transported to the laboratory, and maintained for 1–2 h.

The hepatopancreas tissues were frozen in liquid nitrogen and then stored at −80 °C for physiological assays and RNA extraction. Part of the hepatopancreas tissues were thawed and homogenized at 12,000 rpm for 10 min at 4 °C with 1:9 (*w*/*v*) physiological saline. After centrifugation, the supernatant was diluted and stored in centrifuge tubes for subsequent analysis.

At the 96 h time-point, gills were fixed in 4% paraformaldehyde for 24 h at 4 °C for histological analysis.

### 2.3. Detection of Hemolymph Osmolality

After centrifugation at 10,000× *g* for 20 min at 4 °C, the supernatant of hemolymph samples at each point for three treatment groups was analyzed with a single-channel micro-osmolarity meter (OsmoTECH XT, ADVANCED, Norwood, MA, USA).

### 2.4. Assessment of Physiological Metabolism

The levels of GLU, triglycerides (TG), total protein (TP), and total cholesterol (T-CHO) in the hepatopancreas after 6, 12, 24, 48, and 96 h of salinity stress were determined via commercially available kits (Nanjing Jiancheng Bioengineering Institute, Nanjing, China).

### 2.5. Estimation of Anti-Oxidative Parameter and Immunity Index

The contents of anti-oxidative parameters including total superoxide dismutase (SOD), catalase (CAT), glutathione peroxidase (GSH-Px), glutathione S-transferase (GST), total antioxidant capacity (T-AOC), and malondialdehyde (MDA) content, and the immunity index containing aspartate aminotransferase (AST), alanine aminotransferase (ALT), alkaline phosphatase (AKP), acid phosphatase (ACP), and lysozyme (LZM) in hepatopancreas were determined to evaluate the antioxidant activity under salinity stress using commercially available kits (Nanjing Jiancheng Bioengineering Institute, Nanjing, China).

### 2.6. Histological Analysis

After dehydration, the samples were transparented in xylene, embedded in paraffin, and sectioned (5–6 μm) using a Leica RM2016 Microtomes paraffin slicer (Leica, Weztlar, Germany). Paraffin sections were deparaffinized and rehydrated before staining with hematoxylin–eosin (HE) solution (Nanjing Jiancheng Bioengineering Institute, Nanjing, China). The stained sections were sealed with neutral resin and then visualized under light microscopy (Nikon, Tokyo, Japan). At least six gill filaments were randomly selected for measurement in each visual field, and each gill filament was measured three times. The gill filament diameter was measured by using CaseViewer 2.4 software (3DHISTECH, Budapest, Hungary).

### 2.7. TUNEL Assays

Tissue cell apoptosis was assessed using the terminal-deoxynucleotidyl-transferase-mediated dUTP-biotin nick end labeling (TUNEL) assay following the protocol of the apoptosis detection kit (Wuhan servicebio technology Co., Ltd., Wuhan, China) [31]. Briefly, the sections were deparaffinized and rehydrated, followed by exposure to a protease K working solution. Subsequently, they were incubated with the TUNEL solution and further incubated with 4′,6-diamidino-2-phenylindole (DAPI) to stain the cell nuclei. The sections were then sealed with neutral gum, dried using graded ethanol, and analyzed under a fluorescence microscope (Nikon Eclipse C1, Tokyo, Japan). The live cell nuclei stained with DAPI emitted a blue color upon excitation by ultraviolet light, whereas the positively apoptotic cell nuclei appeared red.

### 2.8. RNA Extraction and cDNA Synthesis

Total RNA was isolated from the hepatopancreases using TRIzol™ reagent (Invitrogen, Waltham, MA, USA). RNA integrity was assessed using 1% agarose gel electrophoresis. Total RNA (1 µg) from different tissues were digested with DNase I (New England Biolabs, Ipswich, MA, USA). First-strand cDNA was synthesized from 1 μg of total RNA using an M-MLV Reverse Transcriptase Kit (Invitrogen, Waltham, MA, USA) following the manufacturer’s instructions.

### 2.9. Real-Time Quantitative PCR (qPCR)

The primers for apoptosis-related genes and reference genes used in this study are listed in Table S1 [32,33,34]. Real-Time qPCR was performed using a StepOnePlus Real-Time PCR System (Applied Biosystems, Foster City, CA, USA) to assess the expression levels of apoptosis-related genes under acute salinity stress. Samples for each qPCR assay contained 1 μL (50 ng/μL) of cDNA, 5 μL of iTaq™ Universal SYBR ^®^ Green Supermix, 0.5 μL of each primer (10 pmol/μL), and 3 μL of double-distilled water to a final volume of 10 μL. The reaction protocol was as follows: 95 °C for 3 min; 35 cycles of 95 °C for 40 s, 60 °C for 45 s, and 72 °C for 30 s; and 72 °C for 10 min for data acquisition, and then 95 °C for 5 s, 60 °C for 30 s, and 95 °C for 15 s to obtain the melt curve. β-actin was selected as the internal reference because of its stable expression.

### 2.10. Correlation Analysis

Pearson’s correlation analysis was performed to explore the correlations between antioxidant parameters and expression levels of key genes in salinity-stressed groups. The heatmap was created in R 4.2.3 with the pheatmap package, and *p*-values less than 0.05 referred to significant differences (*), while values less than 0.01 referred to highly significant differences (**) between two variables.

### 2.11. Statistical Analysis

Differential analysis of hemolymph osmolality, anti-oxidative, and physiological metabolism parameters under different salinity challenges was performed using analysis of variance (ANOVA). Data are presented as the means ± standard error (SE) of three replicates, and the statistical significance is represented by a *p*-value < 0.05. The mRNA abundances of genes were calculated using the 2^−ΔΔCt^ method [35]. All statistical analyses were performed using SPSS v19.0 (SPSS Corp., Armonk, NY, USA).

## 3. Results

### 3.1. Hemolymph Osmolality

**Figure 1:**
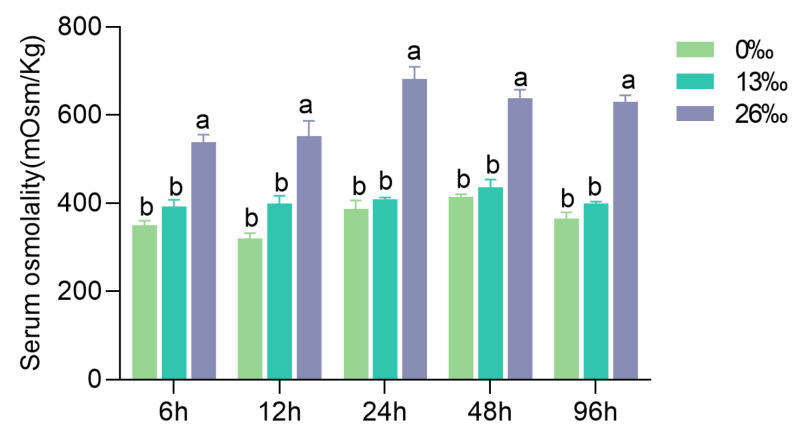
Hemolymph osmolality under different salinity treatments. Different letters indicate significant differences between salinity groups within a given time point (*p* < 0.05). Data are presented as the mean ± standard error (SE) (*n* = 6).

The hemolymph osmolality displayed an increasing trend with water salinity (Figure 1). A significant difference was detected in hemolymph osmotic pressure between the 26‰ group and the 0‰ and 13‰ groups at all the time points (*p* < 0.05). However, there was no difference between the 0‰ and 13‰ salinity groups.

### 3.2. Physiological Metabolism Parameters in Hepatopancreas

**Figure 2:**
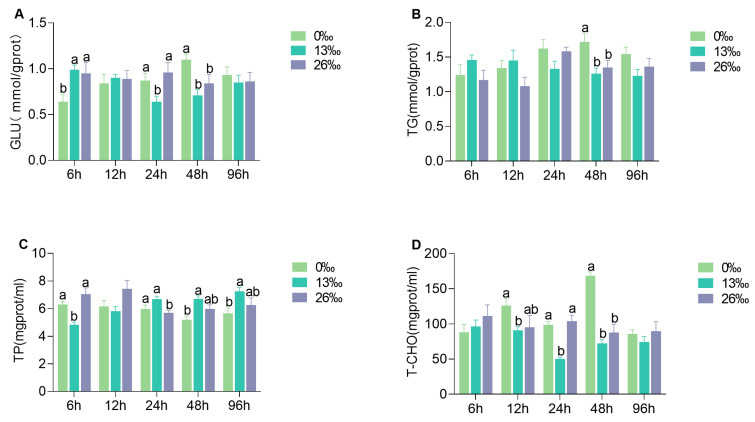
Physiological metabolism parameters in hepatopancreas. (**A**) GLU, glucose; (**B**) TG, triglycerides; (**C**) TP, total protein; and (**D**) T-CHO, total cholesterol. The different lowercase letters represent significant differences within a given time point (*p* < 0.05). Data are presented as the mean ± standard error (SE) (*n* = 6).

Several physiological metabolic parameters of the hepatopancreas were driven by the salinity challenge with significant differentiation at some time points (Figure 2). Significant differences were observed in the GLU contents among the three treatments at 6, 24, and 48 h (*p* < 0.05). At 6 h, the GLU content was significantly lower in the 0‰ group compared to the other groups. Similarly, at 24 h, the 13‰ group displayed a significantly lower GLU level than the other groups (*p* < 0.05). After 96 h, both salinity stress groups exhibited significantly lower GLU levels than the 0‰ group (*p* < 0.05). Furthermore, significant differences in TG contents were detected at 48 h, with significantly higher levels than those in the other two groups (*p* < 0.05). Significant differences in the TP content were found among the three groups at all time-points except 12 h (*p* < 0.05). Specifically, the 13‰ group displayed significantly lower TP levels at 6 h, and the 26‰ group exhibited significantly lower TP levels at 24 h compared to that of the other groups (*p* < 0.05). However, no significant difference was observed between the 0‰ and 26‰ groups at 48–96 h (*p* > 0.05). Following 12–24 h of salinity stress, the 13‰ group showed significantly lower T-CHO contents than the 0‰ group (*p* < 0.05), and the T-CHO content in the 0‰ group was significantly higher than those in the other groups (*p* < 0.05).

### 3.3. Antioxidant Enzyme Activities of Hepatopancreas

**Figure 3:**
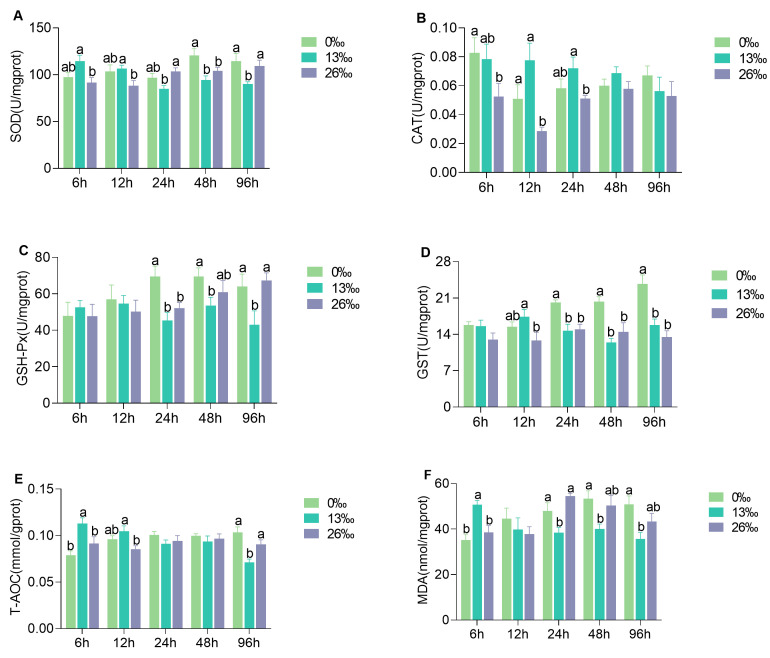
Hepatopancreas antioxidation enzymes activities after salinity stress. (**A**) SOD, superoxide dismutase; (**B**) CAT, catalase; (**C**) GSH-Px, glutathione peroxidase; (**D**) GST, glutathione S-transferases; (**E**) T-AOC, total antioxidant capacity; and (**F**) MDA, malondialdehyde. Different lowercase letters denote significant differences between salinity treatments within a given time point (*p* < 0.05). Data are presented as the mean ± standard error (SE) (*n* = 6).

The antioxidant enzyme activities in the hepatopancreas after salinity stress varied at different time points (Figure 3). The SOD activities of the 13‰ group were significantly higher than those of the 26‰ group at 6 h and 12 h but significantly lower than those of the 26‰ group at 24 and 48 h (*p* < 0.05). At 6, 12, and 24 h, no significant difference was observed between the 0‰ group and the other groups (*p* > 0.05). Significant differences in CAT activities were observed among the three groups at 6, 12, and 24 h (*p* < 0.05). The highest CAT activities were observed in the 13‰ group at 12 and 24 h (*p* < 0.05), but no significant differences were found thereafter (*p* > 0.05). There were no significant differences in GSH-Px activities at 6 or 12 h among the three groups (*p* > 0.05). GSH-Px activities in the 26‰ group increased over time and were significantly higher at 96 h in both the 26‰ and 0‰ groups compared to those in the 13‰ group. Compared to the 0‰ group, GST activity in the 13‰ group and 26‰ group was significantly lower at 24, 48, and 96 h (*p* < 0.05). Significant differences in T-AOC activity were observed among the three groups except at 24 and 48 h. T-AOC activity in the 13‰ group was significantly higher than those in the 26‰ group at 6 and 12 h (*p* < 0.05). However, at 96 h, the T-AOC activity in the 13‰ group was significantly lower than those in the 0‰ and 26‰ groups (*p* < 0.05). Following 24–96 h of salinity stress, the MDA content in the 0‰ group was significantly higher than that in the 13‰ group (*p* < 0.05). The MDA content showed a decreasing trend in the 26‰ group, with no significant difference compared to the 0‰ group (*p* > 0.05).

### 3.4. Immunocompetent Response to Salinity Stress

**Figure 4:**
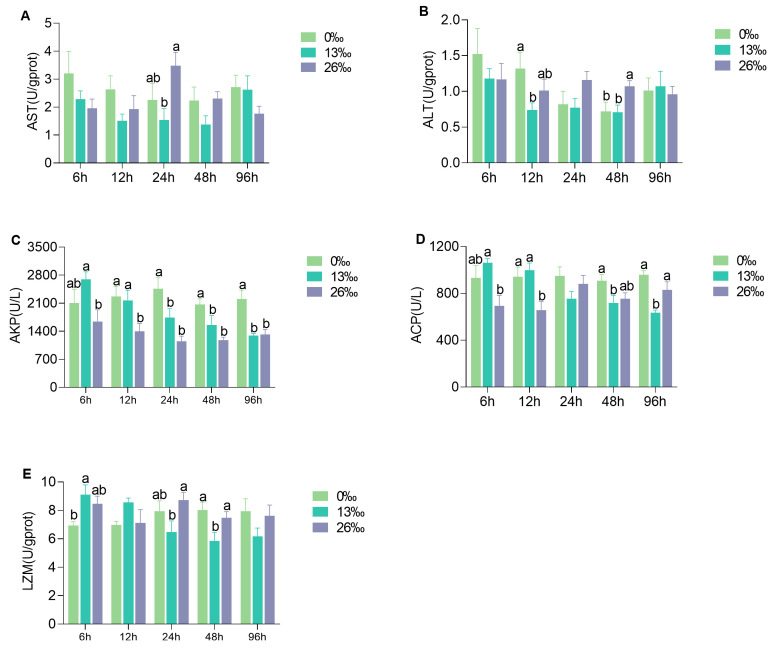
Immunocompetent response of hepatopancreas under different salinity conditions. (**A**) AST, aspartate aminotransferase; (**B**) ALT, alanine aminotransferase; (**C**) AKP, alkaline phosphatase; (**D**) ACP, acid phosphatase; and (**E**) LZM, lysozyme. Different lowercase letters represent significant differences within a given time point (*p* < 0.05). Data are presented as the mean ± standard error (SE) (*n* = 6).

The immunocompetent response of the hepatopancreas to salinity stress exhibited different trends at different time points (Figure 4). After 24 h of salinity stress, AST activity in the 26‰ group was significantly higher than that in the 0‰ group (*p* < 0.05). However, there was no significant difference between the 26‰ and 13‰ groups (*p* > 0.05). At 0‰ salinity, ALT activity was significantly higher after 24 h of exposure compared to that in the 13‰ group (*p* < 0.05). However, after 48 h of exposure, the ALT activity in the 0‰ and 13‰ groups was significantly lower than that in the 26‰ group (*p* < 0.05). Significant differences in AKP activity were observed among the three groups at all time points, with higher activity in the 0‰ group compared to those in the 13‰ and 26‰ groups after 24–96 h of salinity stress (*p* < 0.05). The ACP activity showed a decreasing trend in the 13‰ group, and the opposite trend was observed in the 26‰ group. Significant differences in ACP activity were detected among the three groups at other time points except 24 h (*p* < 0.05). At 12 h, ACP activity was significantly lower in the 26‰ group than in the other groups, and there was no significant difference between the 26‰ and 0‰ groups at 48 and 96 h. The LZM activity in the 13‰ group displayed a decreasing trend, and significant differences were observed among the three groups at 6, 24, and 48 h (*p* < 0.05). At 48 h, the LZM activity was significantly lower in the 13‰ group than in the other groups. However, there were no significant differences between the 0‰ and 26‰ groups at 24–48 h (*p* > 0.05).

### 3.5. Histological Characteristics of Gills

**Figure 5:**
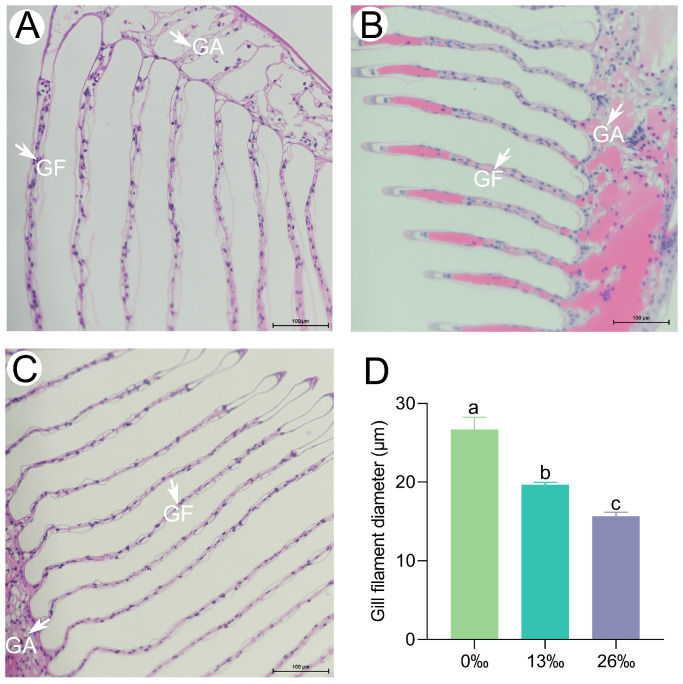
Histological observation on giant freshwater prawn gill tissues. Scale bar = 100 μm. (**A**) 0‰ salinity group; (**B**) 13‰ salinity group; (**C**) 26‰ salinity group; and (**D**) gill filament diameter (GF). GA, gill axis. Different lowercase letters denote significant differences between salinity groups (*p* < 0.05). Data are presented as the mean ± standard error (SE) (*n* = 6).

Significant histological differences were observed in the gills among the different salinity treatments (Figure 5). After 96 h of treatment, the gill filament diameter was significantly smaller in both the 13‰ and 26‰ groups than in the 0‰ group. Overall, salinity stress led to a significant decrease in the gill filament diameter (*p* < 0.05).

### 3.6. Tissue Apoptosis Analysis

**Figure 6:**
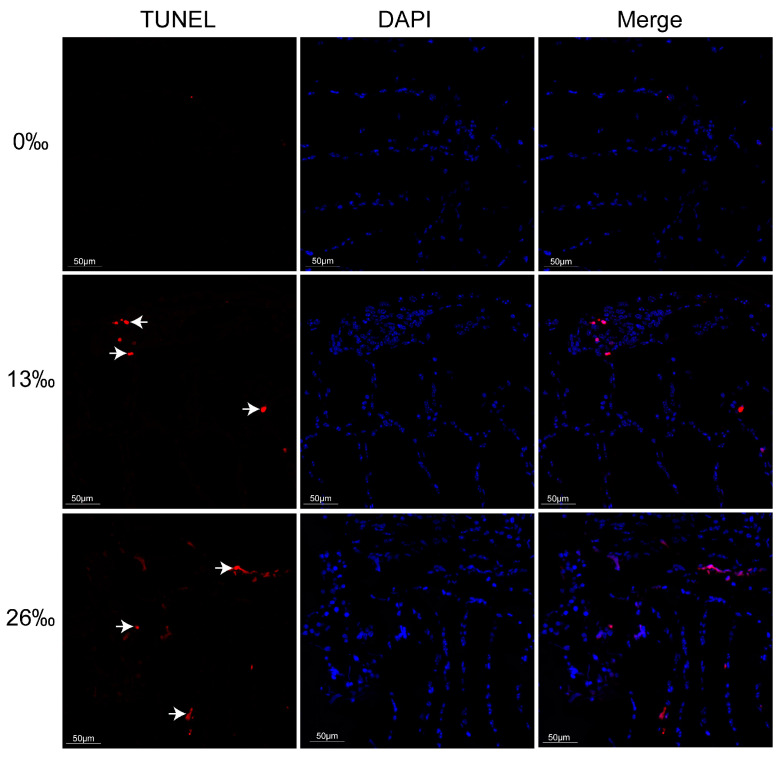
Apoptosis detection in the gill of *M. rosenbergii* at 96 h in different salinity groups (200×). Common live gill cell nuclei appeared as blue signals. TUNEL-positive gill cell nuclei appeared as red signals (arrows). Scale bar = 50 μm.

To further investigate the effects of acute salinity stress on gill tissues, we examined the apoptosis signals using fluorescence microscopy (Figure 6). After 96 h of treatment, no obvious TUNEL signal was observed in the 0‰ group. However, in the 13‰ group, the gill exhibited TUNEL signals in the nuclei, and a more pronounced TUNEL-positive signal was detected in the 26‰ group.

### 3.7. mRNA Expression of Apoptosis-Related Genes

**Figure 7:**
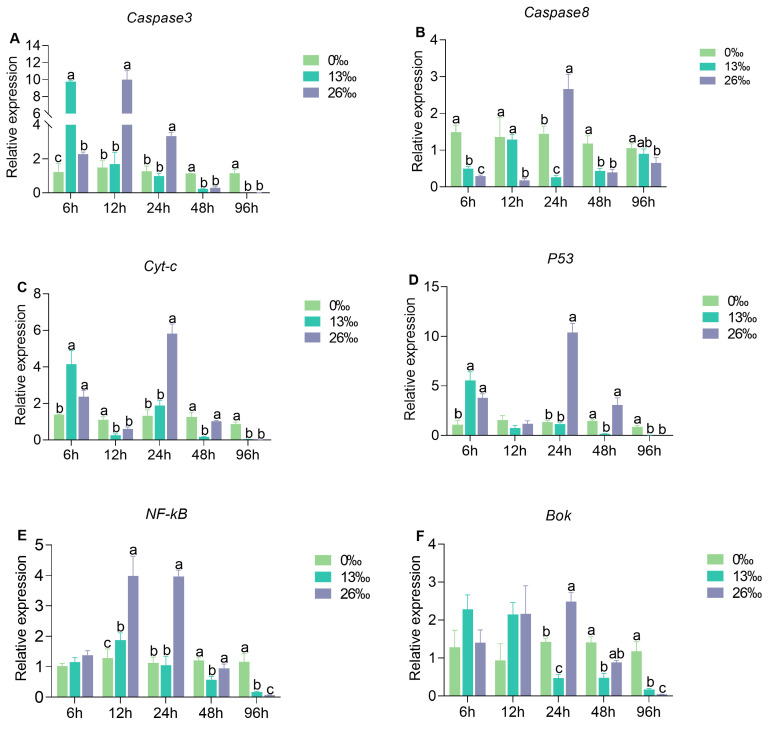
Difference in expression of apoptosis-related genes after different salinity stresses. (**A**) *Caspase 3*, Cysteine-aspartic acid protease 3; (**B**) *Caspase 8*, Cysteine-aspartic acid protease 8; (**C**) *Cyt-c*, Cytochrome c; (**D**) *P53*, tumor suppressor gene; (**E**) *NF-κB*, Nuclear factor kappa-B; and (**F**) *Bok*, B cell lymphoma 2 ovarian killer. Different lowercase letters denote significant differences between salinity treatments within a given time point (*p* < 0.05). Data are presented as the mean ± standard error (SE) (*n* = 6).

The apoptosis-related genes, Cysteine-aspartic acid protease 3 and 8 (*Caspase 3* and *Caspase 3*), Cytochrome c (*Cyt-c*), tumor suppressor gene (*P53*), Nuclear factor kappa-B (*NF-κB*), and B cell lymphoma 2 ovarian killer (*Bok*) exhibited significantly higher expression levels in the 26‰ salinity group compared to the other groups at 24 h, but showed lower levels at 96 h (Figure 7). At 6 h, the transcript levels of *Caspase 3*, *Cyt-c*, and *P53* in 13 and 26‰ groups were significantly higher than those in the control group. However, no significant difference was observed in *NF-κB* and *Bok* expression levels at this time point. After 12 h of salinity treatment, *Caspase 3* and *NF-κB* were significantly upregulated in the 26‰ group, and *Caspase 8* and *Cyt-c* displayed the opposite expression trends. There were no significant differences for *Bok* and *P53* between the groups. *Caspase 8* in the 26‰ salinity group remained low, except at 24 h. After 48 h of exposure to 13‰ salinity, all six genes showed significantly lower expression levels than those in the 0‰ group.

### 3.8. Correlation Analysis between Antioxidant Parameters and Apoptosis-Related Genes

**Figure 8:**
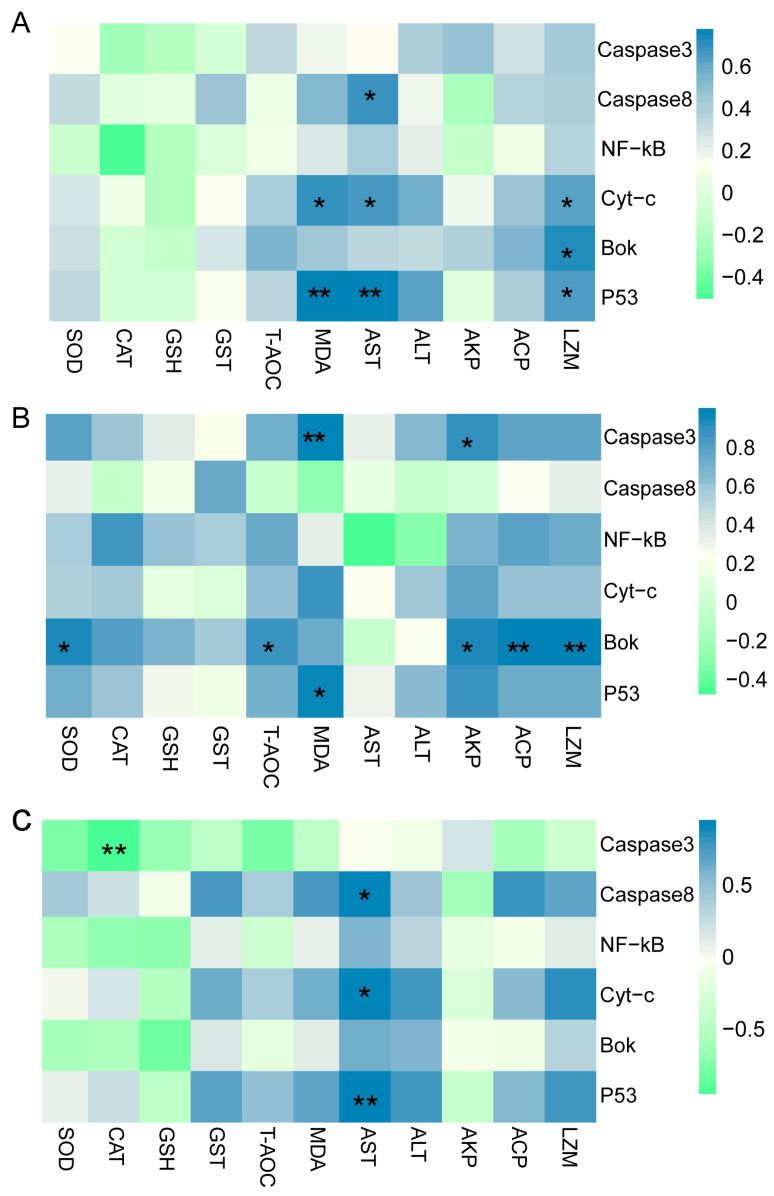
Correlation between antioxidant, immunity enzymatic activities, and key apoptosis-related gene expression levels in salinity stress groups. Correlation in (**A**) salinity stress groups; (**B**) 13‰ salinity; and (**C**) 26‰ salinity; The heatmap shows the Pearson’s correlation coefficient. Steel-blue represents the positive association and pale-green represents the negative association. The color intensity represents the degree of association. * *p* < 0.05, ** *p* < 0.01.

There were significant correlations between apoptosis-related genes *Caspase 3*, *Caspase 8*, *Cyt-c*, *P53*, *NF-kB*, *Bok,* and antioxidant and immune enzymatic activities (Figure 8). A significant positive correlation was observed between the levels of *Cyt-c*, *Bok*, and *P53* mRNA and those of MDA, AST, and LZM. Additionally, *Caspase 8* expression was positively correlated with AST activity. In the 13‰ group, the *Bok* expression level was positively correlated with SOD, T-AOC, AKP, ACP, and LZM activities. The level of *Caspase 3* showed a significant positive correlation with MDA and AKP. The *P53* expression level was only positively correlated with MDA content. In the 26‰ stress group, *Caspase 8*, *Cyt-c*, and *P53* expression levels were positively correlated with AST. However, a significant negative correlation was observed between *Caspase 3* and CAT levels.

## 4. Discussion

Migratory crustaceans often exhibit high tolerance to salinity stress because of their enhanced capacity for hemolymph osmoregulation [5]. The hemolymph maintains the osmotic pressure balance by regulating ion concentration or the expression of Na^+^/K^+^-ATPase genes in gills in response to salinity stress [36]. Our results showed an increase in osmolality with increasing water salinity. However, the osmoregulatory ability greatly declines at the highest salinity and, consequently, the animals approach osmoconformation. The osmoregulation activities under acute salinity stress are in line with earlier studies on decapods [37,38,39]. Similarly, a previous study on *M. rosenbergii* demonstrated that hemolymph osmolality can be maintained at around 420 mOsmolkg-1 at salinity < 18‰ by gradually increasing the salinity, but that the osmotic capacity becomes severely challenged as salinity increases [25]. Our study observed similar changes in hemolymph osmolality, implying that the osmoregulation capacity contributes to the adaption of *M. Rothbergii* to estuarine regions with salinity changes during their early life stages.

Enhanced physiological metabolism produces more energy and substances to help organisms cope with salinity challenges [40]. The hepatopancreas, a key site for storing glycogen, breaks down polysaccharides into monosaccharides such as GLU, releasing energy sources to meet the demands imposed by environmental stresses [41]. Our results revealed significantly higher GLU contents at 6 h but significantly lower levels at 48 h in the 13‰ and 26‰ groups compared to that in the 0‰ group, indicating an extremely high energy consumption after 6 h of salinity exposure. A previous study suggested that GLU can provide energy for osmoregulation in an inshore shrimp under unsuitable salinity conditions [41]. Similarly, both TG and T-CHO contents in the salinity-challenged groups were significantly lower than those in the control group at 48 h. Lipids are considered an essential nutrient for crustaceans, and an appropriate cholesterol level can enhance osmoregulation and salinity stress resistance in the shrimp [42,43]. The decrease in TG and T-CHO reflects their utilization as the main energy source, indicating that more TG and T-CHO in the hepatopancreas tissue were catabolized for osmoregulation.

Acute salinity stress increases the concentration of ROS, resulting in oxidative stress in aquatic animals [19,44,45]. Antioxidant enzymes, such as SOD, CAT, GPx, and T-AOC, play crucial roles in antioxidant defense. SOD eliminates ROS and reduces lipid peroxidation damage, and CAT catalyzes the breakdown of hydrogen peroxide [46]. GSH-Px is considered a key component of the cellular antioxidative system that effectively protects cells against lipid peroxidation damage [47]. MDA levels are commonly used as a marker of cell membrane damage caused by lipid peroxidation [48]. In this study, we observed lower SOD and CAT activities following 6–12 h of higher salinity exposure, indicating that a higher acute salinity stress may inhibit the activity of these enzymes. Similar findings of reduced SOD and CAT activity were reported in a freshwater crab after salinity treatment [36]. We also observed lower activities in GSH-Px and GST from 24 to 96 h after salinity stress, suggesting that GSH-Px and GST may have played a more prominent role in the later phase of acute salinity exposure. T-AOC, an important index for evaluating comprehensive antioxidant capacity, reflects the ability to resist free radical metabolism in response to external stimuli [49]. The higher T-AOC content observed in the 13% salinity group at 6–12 h implies an increased production of free radicals during the early stages of salinity stress. In contrast, the level of MDA decreased first and then increased with the increase in salinity after 24–96 h of salinity stress. Higher-salinity stimulation can lead to decreased MDA levels [50]; therefore, we speculated that the external salinity stress caused extensive oxidative damage when the salinity exceeded the appropriate range for osmoregulation [36].

Environmental stress often triggers non-specific immune responses in aquatic animals [51]. AST, ALT, AKP, and ACP play important roles in immune defense and recognition in crustaceans [52,53]. In our study, we observed higher AST and ALT levels at 24 and 48 h, respectively, in response to 26‰ salinity compared to the 0‰ group. Similarly, higher-salinity treatment was found to increase blood AST and ALT levels in *Clarias gariepinus* [54]. Furthermore, AKP activity maintained a lower level in the 26‰ group than that in 0‰ group throughout the experimental period, which was similar to that in the *Eriocheir sinensis* [36]. The ACP activity in response to 13‰ salinity exposure was significantly lower than that of the 0‰ group near the end of salinity stress (48–96 h), indicating that acute salinity stress affected the immune system of *M. rosenbergii*. In contrast to our results, AKP and ACP activity decreased in response to low salinity stress in the inshore shrimp [55], suggesting opposite trends in AKP and ACP activity between marine crustaceans and freshwater species in response to salinity changes. Notably, AKP and ACP are also important components of the LZM system and function together in the innate immunity of organisms [56,57]. Our study detected higher LZM activity in the 26‰ group than in the 13‰ group at 24 h and 48 h, which is consistent with the findings in a coastal fish, where LZM activity recovered to the same level as that of the control group at 48 h [56]. These results indicate that *M. rosenbergii* exhibits a stronger tolerance to high salinity than lower salinity.

As respiratory and osmoregulatory organs, gills exhibit rapid responses to salinity stress, primarily through significant morphological changes, such as gill filament shrinking and deformation, reductions in gill cilia, and cell apoptosis [58]. In our study, we observed gill filament elongation during exposure to higher-salinity conditions. This finding is consistent with previous studies on an estuary crab and a congeneric freshwater prawn, in which the gill filament diameter became thicker and thinner in response to hypo-salinity and hyper-salinity stress, respectively [59,60]. These results suggest that crustaceans living in freshwater or saltwater environments exhibit similar patterns in response to changes in external salinity, such as alterations in gill filament morphology [61]. Excessive environmental stress can stimulate the overproduction of ROS and subsequently induce cell apoptosis [51]. In this study, a more pronounced TUNEL-positive signal was detected in the higher-salinity group (26‰), which may have been attributed to the extensive oxidative damage caused by acute external salinity changes, leading to cell apoptosis.

The prolonged oxidative stress promotes cell death by activating the apoptotic pathway [62]. In our study, the expression levels of apoptosis-related genes, including *Caspase 3*, *Caspase 8*, *Cyt-c*, *P53*, *NF-κB*, and *Bok*, showed an initial increase followed by a decrease under salinity exposure. Down-regulation of those apoptosis-related genes indicates that the late stage of the apoptosis process was reached at the end of the high-salinity exposure experiment. *Caspase 3* and *Caspase 8* are crucial molecules in apoptosis, with *Caspase 8* initiating the caspase cascade and activating downstream *Caspase 3* to induce apoptosis [63]. Caspase 3 activity is first detectable early in apoptosis, continues to increase as cells undergo apoptosis, and rapidly declines during the late stages of apoptosis [64]. The lowest *Caspase 3* level in the 26‰ group suggested that apoptosis reached its final stage after 96 h. Interestingly, we found a significant negative relationship between *Caspase 3* transcription abundance and CAT activity in the 26‰ salinity group. The active Caspase 3 can inhibit CAT activity and induce proteolysis of the CAT protein [65]. Therefore, we hypothesize that *Caspase 3* and CAT may play antagonistic roles in response to higher-salinity challenges.

The activation of caspases, induced by the release of *Cyt-c*, is a crucial step in cell apoptosis. As a transcription factor, the *Cyt-c* mRNA expression influences apoptosis activation in the mitochondrial pathway [66]. In our study, we found that *Cyt-c* and *P53* expression was significantly related to MDA and AST levels under salinity stress, indicating that high salinity induced apoptosis in gills through the mitochondrial pathway. *P53* has been well established to play a vital role in cell death by regulating the apoptosis pathway [67]. The significant up-regulation of *P53* in a marine crab at low salinity from 3 h to 24 h is opposite yet analogous to our findings, suggesting a conserved role of P53 in responding to salinity changes among different crustaceans [68]. Furthermore, *P53* actively participates in the cell cycle and apoptosis process by promoting the transcriptional regulation of downstream target genes [69].

*NF-κB* is a highly conserved transcription factor, and activation of the *NF-κB* signaling pathway induced by environmental stressors usually triggers the expression of downstream genes involved in various cellular events, including apoptosis [70]. The NF-κB signaling pathway was enriched, and *NF-κB* expression was up-regulated under salinity and temperature stresses in a coral [71].

*Bok* is a proapoptotic protein that promotes cell death by interacting with specific anti-apoptotic proteins. Its overexpression has been reported to promote the repair of oxidative damage caused by hydrogen peroxide [72]. Based on our results, lower *Bok* expression levels may inhibit the oxidative damage repair. In the 13‰ group, *Bok* expression level was significantly correlated with SOD, T-AOC, ACP, AKP, and LZM, suggesting that the oxidative damage and inflammation caused by salinity stress may have stimulated damage repair mechanisms by inducing *Bok* expression; however, this requires further investigation.

## 5. Conclusions

**Figure 9:**
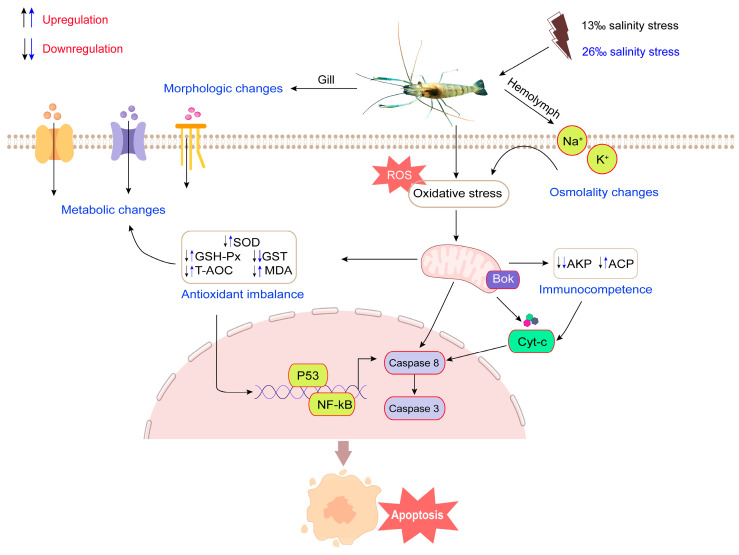
Plausible mechanism of responding to acute salinity stress in *M. rosenbergii*. Acute salinity stress leads to changes in hemolymph osmotic pressure, causing changes in gill morphology and structure. To adapt to the salinity changes, the organism’s metabolic capacity (GLU, TG, TP, and T-CHO level) is enhanced and activities of some enzymes involved in antioxidant and immune processes are changed, leading to oxidative stress in mitochondria, activation of apoptosis-related pathways, initiation of the expression of related genes (*Caspase 3*, *Caspase 8*, *Cyt-c*, *P53*, *NF-κB*, and *Bok*), and the triggering of apoptosis in tissues. The upward arrow represents increasing enzyme activities and the downward arrow represents decreasing enzyme activities.

Acute salinity stress induces an increase in hemolymph osmotic pressure, triggers antioxidant responses, and elicits immune stress, leading to structural damage and apoptosis of *M. rosenbergii* gill tissue (Figure 9). These findings provide valuable insights into the molecular basis of the response to salinity stress, which will improve aquaculture and management practices of *M. rosenbergii* and enhance our understanding of the potential molecular mechanisms underlying the adaptation to acute salinity stress in crustaceans.

## Data Availability

The data are contained within the article and Supplementary Materials.

## Supplementary Materials

The following supporting information can be downloaded at https://www.mdpi.com/article/10.3390/antiox12101836/s1, Table S1: qRT-PCR primers used in the study.
